# Patterns in hydraulic architecture from roots to branches in six tropical tree species from cacao agroforestry and their relation to wood density and stem growth

**DOI:** 10.3389/fpls.2015.00191

**Published:** 2015-03-31

**Authors:** Martyna M. Kotowska, Dietrich Hertel, Yasmin Abou Rajab, Henry Barus, Bernhard Schuldt

**Affiliations:** ^1^Plant Ecology and Ecosystems Research, Albrecht von Haller Institute for Plant Sciences, University of Göttingen, GöttingenGermany; ^2^Faculty of Agriculture, Tadulaku University, PaluIndonesia

**Keywords:** shade tree, hydraulic conductivity, wood density, aboveground productivity, foliar nitrogen, perhumid climate, vessel diameter

## Abstract

For decades it has been assumed that the largest vessels are generally found in roots and that vessel size and corresponding sapwood area-specific hydraulic conductivity are acropetally decreasing toward the distal twigs. However, recent studies from the perhumid tropics revealed a hump-shaped vessel size distribution. Worldwide tropical perhumid forests are extensively replaced by agroforestry systems often using introduced species of various biogeographical and climatic origins. Nonetheless, it is unknown so far what kind of hydraulic architectural patterns are developed in those agroforestry tree species and which impact this exerts regarding important tree functional traits, such as stem growth, hydraulic efficiency and wood density (WD). We investigated wood anatomical and hydraulic properties of the root, stem and branch wood in *Theobroma cacao* and five common shade tree species in agroforestry systems on Sulawesi (Indonesia); three of these were strictly perhumid tree species, and the other three tree species are tolerating seasonal drought. The overall goal of our study was to relate these properties to stem growth and other tree functional traits such as foliar nitrogen content and sapwood to leaf area ratio. Our results confirmed a hump-shaped vessel size distribution in nearly all species. Drought-adapted species showed divergent patterns of hydraulic conductivity, vessel density, and relative vessel lumen area between root, stem and branch wood compared to wet forest species. Confirming findings from natural old-growth forests in the same region, WD showed no relationship to specific conductivity. Overall, aboveground growth performance was better predicted by specific hydraulic conductivity than by foliar traits and WD. Our study results suggest that future research on conceptual trade-offs of tree hydraulic architecture should consider biogeographical patterns underlining the importance of anatomical adaptation mechanisms to environment.

## Introduction

The water transport pattern in trees is mainly determined by the plant hydraulic architecture, i.e., the spatial distribution of various xylem properties from roots to branches of a tree individual (McCulloh et al., 2010). The hydraulic efficiency of different compartments along the root-to-leaf flow path can be described by the sapwood area-specific hydraulic conductivity (*K*_S_), which is directly related to the hydraulic resistance of a given position (Tyree and Ewers, 1991; McElrone et al., 2004). According to the Hagen–Poiseuille law, even a small increase in mean vessel diameter causes an exponential increase of *K*_S_. This anatomical pattern represents the most economical way for a woody plant to enhance the path-length hydraulic conductivity. Independently of the efficiency of the hydraulic system water is transported in a metastable state below its vapor pressure in vascular plants, which makes them vulnerable to the formation of gas embolism. This can impair the transport of water from the soil to the leaves. Particularly wide vessels are not only most conductive but also most prone to the risk of hydraulic failure in form of xylem embolisms (Maherali et al., 2006; Awad et al., 2010; Cai et al., 2010; Hajek et al., 2014), resulting in a trade-off between hydraulic efficiency and cavitation resistance. As one of the basic organizing principles of tree hydraulic architecture it has been postulated that the mean vessel diameter in the xylem tissue generally decreases acropetally from roots to branches (‘vessel tapering’: Baas, 1982; Tyree and Zimmermann, 2002; Anfodillo et al., 2013). This principle has stimulated several conceptual models on plant hydraulic architecture during the past 15 years. They state that whole-plant hydraulic conductance dependent on distance to ground in support of the model by West et al. (1999) and Murray’s law (McCulloh et al., 2003). Consistent with these predictions it has indeed commonly been observed that the largest vessels along the water flow path are found in roots of trees from temperate or Mediterranean environments (Martinez-Vilalta et al., 2002; Pratt et al., 2007; Domec et al., 2009). However, recent studies in tropical forests in South America (Machado et al., 2007; Fortunel et al., 2013) and Indonesia (Schuldt et al., 2013) have produced contradictory results regarding the paradigm of continuous vessel tapering. Schuldt et al. (2013) supposed that mechanisms reducing cavitation risk may not have been evolved in these moist or perhumid environments where drought stress is normally not apparent.

Forested perhumid regions particularly in the tropics are underrepresented in studies so far and are moreover converted rapidly. Worldwide approximately 27.2 million ha of humid tropical forests have been cleared between 2000 and 2005 (Hansen et al., 2008) mainly for agricultural land use (Achard et al., 2002; FAO and JRC, 2012). In South-East Asia, a common driver of deforestation is the conversion of natural forests into cacao (*Theobroma cacao*) agroforestry systems. Cacao is native to tropical South America (Motamayor et al., 2008) and represents one of the commercially most important perennial cash crops worldwide. Traditionally cacao trees are planted under selectively thinned primary or older secondary forest in Indonesia, but nowadays cultivation is shifting to non-shaded monocultures or agroforests with introduced fast-growing legume tree species such as *Gliricidia sepium* to increase short-term income (Rice and Greenberg, 2000). Shade trees in cacao plantations enhance functional biodiversity, carbon sequestration, soil fertility and drought resistance and provide microclimatic benefits such as increased humidity and buffering temperature extremes (Schroth and Harvey, 2007; Tscharntke et al., 2011).

Considering the ecological relevance of the anatomical hydraulic properties described above, it is important to note that systematic studies on the ecological wood anatomy and hydraulic architecture of cacao and shade tree species are lacking so far. This is all the more unsatisfying since tropical agroforestry crop and shade tree species often originate from different biomes and possess distinct drought adaptations, but it is unknown so far if this implies differences in the hydraulic strategy of those crop and shade tree species. It is therefore unknown whether cacao and shade tree species in the agroforestry systems with different biogeographical origin have developed similar hydraulic properties as the tree species of the natural forest replaced by those.

A high aboveground biomass (AGB) production (including high crop yield) has been related to several plant functional traits like high stem hydraulic efficiency, high foliar nitrogen content, or low stem wood density (WD; Brodribb et al., 2002; Tyree, 2003; Zhang and Cao, 2009; Hoeber et al., 2014). Thereby low WD implying lower hydraulic safety is found to be associated with fast tree growth (Enquist et al., 1999; King et al., 2005; Poorter et al., 2010), while species with dense wood are considered to be more resistant to xylem cavitation due to the commonly assumed relation between WD and conduit size and thus xylem wall thickness and resistance to cell wall implosion under negative pressure (Jacobsen et al., 2005). Consequently, species with dense wood should show higher hydraulic safety at the cost of lower productivity (Meinzer et al., 2003; Bucci et al., 2004). Nevertheless, several studies, particularly from tropical environments, found WD decoupled of hydraulic efficiency traits and growth performance (Zhang and Cao, 2009; Russo et al., 2010; Fan et al., 2012; Schuldt et al., 2013). It would therefore be interesting to assess whether hydraulic properties and WD are related to the aboveground performance of crop and shade tree species in cacao agroforests.

In this study, we examined the inter-relationship between sapwood area-specific hydraulic conductivity of the root, stem, and branch xylem tissue with wood anatomical traits along the water flow path across six common cacao agroforestry tree species with different biogeographical origins from either seasonally dry or perhumid tropical environments growing in cacao agroforests in Central Sulawesi (Indonesia). We moreover wanted to relate aboveground growth performance to hydraulic efficiency, stem WD, foliar nitrogen content and foliar δ^13^C of these species. We hypothesized (i) that – in contrast to temperate tree species – the largest vessels along the water flow path are found in the stem xylem and not in the roots, (ii) that stem xylem hydraulic properties are unrelated to stem WD, and (iii) that aboveground productivity across species is positively related to vessel size and hydraulic conductivity.

## Materials and Methods

### Study site, Species, and Sampling

The study was carried out in a cacao agroforestry located in the Kulawi Valley, Bolabapu District, Central Sulawesi, Indonesia (S 01°55.9^′^ E 120°02.2^′^, elevation 571 m above see level) in May 2012. The climate of the study region is perhumid without a distinct dry season. Mean annual temperature recorded for the study area by Moser et al. (2010) is 25.5°C and mean annual precipitation is 2092 mm between 2002 and 2006. For the study, a cacao agroforestry plot with multi-species shade tree layer was selected from a larger number of preselected cacao agroforestry plots of a different investigation that were found to be representative in terms of management, aboveground structure and topographical patterns for this region. Caution was taken during the selection process that the plot was far enough above the groundwater table to guarantee that the trees had no direct access to this water source. All trees in the agroforest were planted simultaneously around 25 years ago.

*Theobroma cacao* L. (Malvaceae) originating from rainforests of lowland northern South America and five common shade tree species were studied: *Leucaena leucocephala* (Lam.) de Wit and* Gliricidia sepium* (Jacq.) Steud. (both Fabaceae), which are introduced species from seasonal dry forest areas of Central America. The three other species represent native origins: *Gnetum gnemon* L. (Gnetaceae), the short-term drought-tolerating* Erythrina subumbrans* (Hassk.) Merill (Fabaceae) and the strictly perhumid species *Durio zibethinus*. Murr. (Malvaceae). In the following we have grouped the species according to their drought tolerance as perhumid (*T. cacao, D. zibethinus, G. gnemon*) and seasonal (*G. sepium*, *L. leucocephala, E. subumbrans*). All species have diffuse-porous wood with *G. gnemon* being a gymnosperm bearing vessels structurally similar to angiosperms (Carlquist, 1994; Fisher and Ewers, 1995). We chose six tree replicates of each species with a diameter and height representative for the whole agroforestry (**Table 2**). For each tree three sun-exposed upper-crown branches and three topsoil root segments (diameter 6–14 mm; length 25–35 cm) were collected as well as one stem core of 5 cm length per tree taken with an increment corer (Haglöf, Långsele, Sweden) at 130 cm stem height. To ensure species identity the roots were traced back to the tree stem. In order to avoid microbial growth in the extracted tree organs, samples were stored in polyethylene tubes filled with water containing a sodium–silver chloride complex (Micropur Katadyn, Wallisellen, Switzerland). The samples were kept cool at 4°C and the conductivity measurements took place not more than 7 days after collection.

**Table 1:** List of major variables with definition and units employed.

Symbol	Unit	Definition
H	cm	Tree height
DBH	cm	Diameter at breast height
AGB	kg	Aboveground biomass
BAI	cm^2^ yr^-1^	Basal area increment
WD	g cm^-3^	Wood density
*d*	μm	Vessel diameter
*d*_h_	μm	Hydraulically weighted vessel diameter
VD	n mm^-2^	Vessel density
*A*_lumen_	%	Relative vessel lumen area (lumen to sapwood area ratio)
*A*_cross_	mm^2^	Branch cross sectional area
*A*_xylem_	mm^2^	Branch sapwood area
*K*_S_^emp^	kg m^-1^ MPa^-1^ s^-1^	Empirical sapwood area-specific hydraulic conductivity
*K*_S_^theo^	kg m^-1^ MPa^-1^ s^-1^	Theoretical sapwood area-specific hydraulic conductivity
*K*_L_^emp^	10^-4^ kg m^-1^ MPa^-1^ s^-1^	Empirical leaf area-specific hydraulic conductivity
*K*_L_^theo^	10^-4^ kg m^-1^ MPa^-1^ s^-1^	Theoretical leaf area-specific hydraulic conductivity
*N*_leaf_	g kg^-1^	Foliar mass-specific nitrogen content
SLA	cm^2^ g^-1^	Specific leaf area
HV	10^-4^ m^2^ m^-2^	Sapwood to leaf area ratio (Huber value)
δ^13^C	‰	Carbon isotope signature

**Table 2:** Tree height (H), diameter at breast height (DBH), wood density (WD), aboveground biomass (AGB), and basal area increment (BAI) of the six tree species in cocoa agroforests.

	Species	Code	*n*	H (m)	DBH (cm)	WD (g cm^-3^)	AGB (kg)	BAI (cm^2^ yr^-1^)
**Perhumid**
	*Theobroma cacao*	Th_ca	6	5.83 ± 0.37	11.36 ± 0.45	0.398 ± 0.007	16.89 ± 2.14	6.51 ± 1.92
	*Durio zibethinus*	Du_zi	6	14.10 ± 1.44	25.56 ± 4.03	0.430 ± 0.019	230.65 ± 72.72	67.99 ± 20.03
	*Gnetum gnemon*	Gn_gn	6	12.40 ± 0.30	18.73 ± 1.63	0.591 ± 0.013	131.80 ± 20.71	28.34 ± 9.71
**Seasonal**
	*Gliricidia sepium*	Gl_se	6	10.90 ± 0.56	11.68 ± 0.51	0.601 ± 0.029	45.75 ± 3.67	19.02 ± 4.83
	*Leucaena leucocephala*	Le_le	6	13.75 ± 2.07	36.30 ± 8.61	0.609 ± 0.010	888.20 ± 320.79	87.61 ± 28.87
	*Erythrina subumbrans*	Er_su	6	10.06 ± 0.58	33.05 ± 2.29	0.273 ± 0.008	162.03 ± 20.88	10.18^∗^

Shown values are means ± SE and the number of investigated tree individuals. ^∗^For BAI, however, only three tree individuals of *Gnetum gnemon*, two of *L. leucocephala*, and one of *E. subumbrans* were available (see Materials and Methods).

### WD, AGB, and Productivity

Wood density, defined as oven-dry weight over wet volume, was measured for each stem core. The fresh volume of each sample was determined by Archimedes’ principle. Samples were then oven dried for 48 h at 105°C and dry mass recorded.

Aboveground biomass of the trees was calculated using the allometric equation of Chave et al. (2005) for tropical wet stands as: AGB = exp [-2.187 + 0.916 × ln (WD × DBH^2^ × H)], where AGB is the estimated aboveground biomass (kg), DBH the trunk diameter at 130 cm height (cm), H the total tree height (m), and WD the stem wood density (g cm^-3^). Since we obtained proper data on tree height only at the beginning of the study, we used stem basal area increment (BAI, cm^2^ yr^-1^) determined over a period of 12 months using dendrometer tapes (UMS GmbH, München, Germany) as indicator for aboveground productivity. However, it has been shown that AGB increment and BAI are very closely related in tropical trees (Hoeber et al., 2014). For *T. cacao, G. sepium and D. zibethinus* six tree replicates were monitored, whereas data from just three *G. gnemon*, two *L. leucocephala* and one of* E. subumbrans* were available for BAI.

### Leaf Morphological and Chemical Properties

From each branch segment harvested for the hydraulic and anatomical measurements, all distal leaves were stripped off and oven-dried at 70°C for 48h to determine leaf dry weight. Specific leaf area (SLA, cm^2^ g^-1^) values were determined using data from nine additional branches per species where leaf surface areas were measured with the WinFOLIA software (Régent Instruments, Quebec, QC, Canada). Total leaf area per branch segment (*A*_L_, m^2^) was calculated by dividing dry weight through species-specific SLA values. Subsequently, leaf samples were grounded and analyzed for their foliar concentrations of C and N and for their foliar signatures of δ^13^C in the leaf bulk tissue with a Delta plus isotope mass spectrometer (Finnigan MAT, Bremen, Germany), a Conflo III interface (Thermo Electron Coorperation, Bremen, Germany) and a NA2500 elemental analyzer (CE-Instruments, Rodano, Milano, Italy) using standard δ notion: δ = (*R*_sample_/*R*_standard_ - 1) × 1000 (‰) in the laboratory for stable isotope measurements (KOSI) at the University of Göttingen.

### Empirical Conductivity Measurements

Hydraulic conductivity of one to three root and branch segments per tree was empirically measured using the method described by Sperry et al. (1988). In total, 44 root and 39 branch segments were analyzed (mean root segment length ± SE: 291 ± 7.0 mm and diameter: 7.87 ± 0.25 mm; mean branch segment length: 308 ± 4.3 mm, and diameter: 9.12 ± 0.29 mm). All segments were recut under water with a razor blade, small lateral roots, and branches cut-off and sealed with quick-drying superglue (Loctite 431, Henkel, Düsseldorf, Germany) and activator (Loctite 7452 Aktivator, Henkel, Düsseldorf, Germany) that function on wet materials. Afterward, segments were attached under water to the tubing system of the conductivity apparatus, where the pressure difference of 6 kPa was generated by a 60 cm high water column. De-ionized water with a sodium–silver chloride complex (16 μg L^-1^ Ag, 8 mg L^-1^ NaCl, Micropur katadyn, Wallisellen, Switzerland) was used as measuring solution in order to avoid microbial growth in the tubing system, a common problem in tropical environments. While comparing our data with conductivities determined by other solutions, it has to be considered that different perfusion solutions can affect hydraulic conductivity (Espino and Schenk, 2011). The solution was passed through a 0.2 μm membrane filter (Maxi Capsule, Pall Corporation, USA) and each sample measured three times in row and flushed with the measuring solution for 5 min at 120 kPa in between each measurement to remove potential emboli. The hydraulic conductivity (*K*_h_^emp^, kg m s^-1^MPa^-1^) was calculated as *K*_h_ = (ΔV/Δt) × (l/ΔP), where l is the length of the segment (m), ΔP the pressure difference applied to the segment (MPa), ΔV the amount of water flowing out of the segment (kg), and Δt the time interval of measurement (s).

Segments of the branches and roots used for conductivity measurements were planed with a sliding microtome (G.S.L.1, WSL, Birmensdorf, Switzerland) to obtain high-quality top view images with a stereo-microscope (SteREOV20, Carl Zeiss MicroImaging GmbH, Göttingen, Gemany) and total cross-sectional (*A*_cross_, mm^2^) and xylem cross-sectional area (*A*_xylem_, mm^2^) analyzed with ImageJ (v1.44p). Subsequently, for each species a regression analysis between *A*_cross_ and *A*_xylem_ was carried out (Table A1). Empirical sapwood area-specific hydraulic conductivity (*K*_S_^emp^, kg m^-1^ MPa^-1^ s^-1^) was calculated by dividing *K*_h_^emp^ by the calculated mean xylem cross-sectional area without pith and bark by applying the species-specific regression coefficients, and empirical leaf area-specific hydraulic conductivity (*K*_L_^emp^, kg m^-1^ MPa^-1^ s^-1^) by dividing *K*_h_ by the total supported leaf area (*A*_L_).

### Vascular Anatomy

For the cross-sectional xylem anatomical analysis, 3 cm of the basipetal end of each root or branch segment used for empirical conductivity measurements was stained with safranin (1% in 50% ethanol, Merck, Darmstadt, Germany) and 10–20 μm semi-thin disks cut with a sliding microtome (G.S.L.1, WSL, Birmensdorf, Switzerland). For stem wood anatomy the outermost 4 cm of the increment core were used. Photographs of the cross-sectional cuts were taken with a stereo-microscope with an automatic stage equipped with a digital camera (SteREOV20, Carl Zeiss MicroImaging GmbH, Göttingen, Gemany) at 100× magnification. Per sample, 32 up to 107 single images were stitched together to obtain the whole cross-sectional area. Image processing was done with Adobe Photoshop CS6 (version 13.0.1, Adobe Systems Incorporated, USA) and ImageJ^1^ (version 1.47) using the particle analysis-function for estimating vessel density (VD, n mm^-1^), the idealized vessels diameter (*d*) from major (*a*) and minor (*b*) vessel radii using the equation given by White (1991) as *d* = [(32 × (a × b)^3^)/(a^2^ + b^2^)]^1/4^, and cumulative vessels lumen area (*A*_lumen_, m^2^). Single vessel diameters (*d*) were used to calculate the hydraulically weighted vessel diameter (*d*_h_) according to Sperry et al. (1994) as *d*_h_ = ∑*d*^4^/∑*d*^5^. For these measurements all vessels of a cross section were analyzed, yielding 110 to 3,600 measured vessel per species and organ. The theoretical hydraulic conductivity (*K*_h_^theo^) of a segment was calculated based on Hagen–Poiseuille’s law as *K*_h_^theo^ = ((π × ∑r^4^)/8η)×ρ, where *r* is the vessel radius, η the viscosity (1.002 × 10^-3^ Pa s) and *ρ* the density of water (998.2 kg m^-3^), both at 20°C. Theoretical sapwood area-specific hydraulic conductivity (*K*_S_^theo^, kg m^-1^ MPa^-1^ s^-1^) was obtained from *K*_h_^theo^ by dividing through the microscopically determined xylem cross-sectional area without bark and pit, and theoretical leaf area-specific hydraulic conductivity (*K*_L_^theo^, kg m^-1^ MPa^-1^ s^-1^) by division of *K*_h_^theo^ by the total supported leaf area (*A*_L_).

### Statistical Analyses

A principal-component analysis (PCA) was done to evaluate how aboveground growth performance, wood anatomical and leaf traits are associated among each other using the package CANOCO, version 4.5 (Biometris, Wageningen, the Netherlands). The matrix species factors were lumen area (*A*_lumen_), VD, hydraulically weighted vessel diameter (*d*_h_), stem basal increment (BAI), empirical (*K*_S_^emp^) as well as theoretical hydraulic conductivity (*K*_S_^theo^). All other statistical calculations were done with the R software package, version 3.1.0 (R Development Core Team, 2014). Pearson correlations were calculated for all pairwise combinations of wood anatomical properties, WD, and hydraulic traits. In case of non-linear relationships where the data are presented on a log-linear scale, the data were log10 transformed to achieve normal distribution before further statistical analyses were conducted. Comparisons of hydraulic and leaf traits among organs were conducted using mixed linear models (lme, package: ‘nlme’ and lm package: ‘stats’) with species as random factor to account for pseudoreplication. Predicted random effects and residuals of the models were checked for normal distribution and homoscedasticity using diagnosis plots and dependent variables were log-transformed and/or variance functions (varIdent or varExp) were used (Pinheiro and Bates, 2000) when necessary. Subsequently, multiple comparison tests between group means were tested *post hoc* with Tukey HSD tests (glht package: ‘multcomp’). In case of heteroscedasticity an adjusted statistical framework for simultaneous inference and robust covariance estimators (Herberich et al., 2010) was used to account for different variances between groups. To test the best predictor for aboveground growth performance we applied stepwise backward model selection (step.AIC, package: ‘MASS’) to identify the most parsimonious model, defined as the model with the lowest AIC (Akaike information criterion) score (Burnham and Anderson, 2002) including *K*_S_^theo^, WD, N_leaf_, δ^13^C as well as species affiliation (whether it is perhumid or seasonal) and their interactions as explanatory variables.

## Results

### Tree Size and Aboveground Growth Performance

**FIGURE 1:**
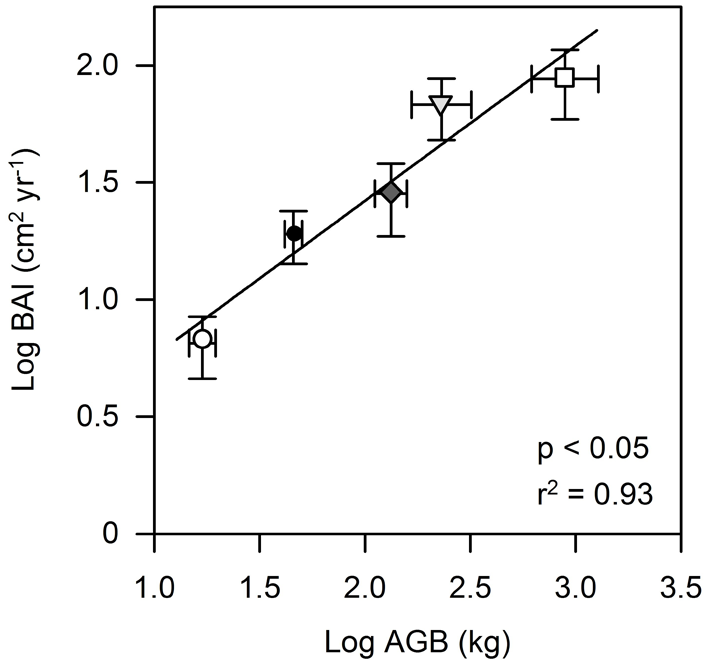
**Relationship between stem basal area increment (BAI) of cacao and four shade tree species and aboveground biomass (AGB).** Each symbol represents mean values for each tree species (○ Th_ca; ∇ Du_zi; ⧫ Gl_se; □ Le_le; ● Gn_gn). Error bars indicate 1 SE.

The variability in mean AGB between the studied species was high, ranging between 16.9 kg in *T. cacao* and 888.2 kg in *L. leucocephala* reflecting marked differences in height and diameter between the pruned *T. cacao* and* G. sepium*, and the other four shade tree species (**Table 2**). *L. leucocephala* was on average more than two times higher and larger compared to *T. cacao*; the other four species ranged between these two extremes (**Table 2**) even though all trees were planted at the same time. Stem WD varied by a factor of two across the six tree species with *E. subumbrans* showing the lowest WD and *G. sepium* and *L. leucocephala* showing the highest WD values. AGB was found to be a very good predictor for the annual BAI amongst all species (**Figure 1**). BAI numbers were thus very different across the six tree species and ranged from 6.5 and 10.2 cm^2^ yr^-1^ in *T. cacao* and *E. subumbrans*, respectively, to 68 and 88 cm^2^ yr^-1^ in *D. zibethinus* and *L. leucocephala*, respectively (**Table 2**).

### Changes in Hydraulic Conductivity Along the Water Flow Path

**FIGURE 2:**
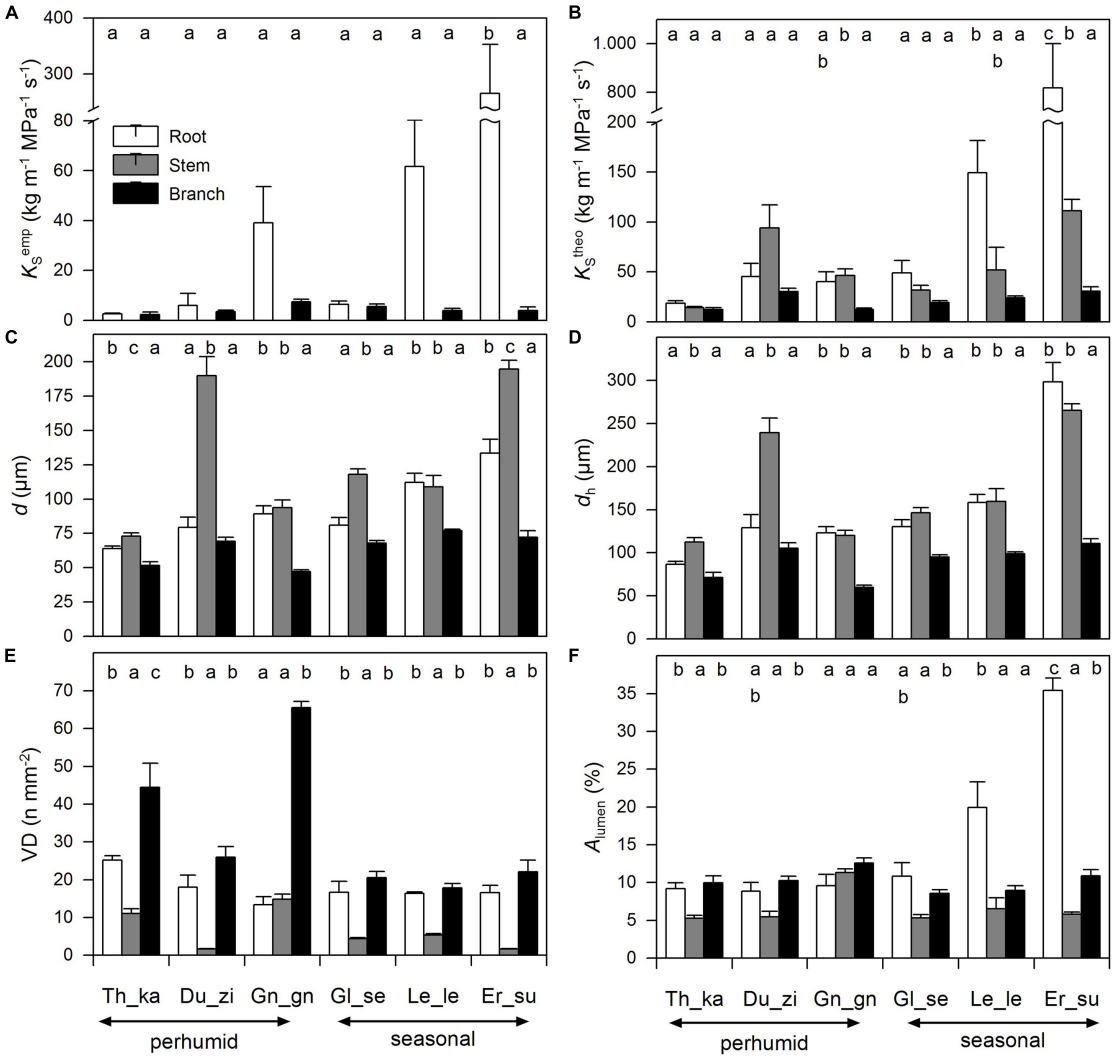
**Hydraulic characteristics – **(A)** empirical sapwood area-specific hydraulic conductivity (*K*_**S**_^**emp**^), **(B)** theoretically calculated sapwood area-specific hydraulic conductivity (*K*_**S**_^**theo**^), **(C)** vessel diameter (*d*), **(D)** hydraulically weighted vessel diameter (*d*_**h**_), **(E)** vessel density (VD), and **(F)** relative lumen area (*A*_**lumen**_) – of six cacao agroforestry species (Th_ca; Du_zi; Gn_gn; Gl_se; Le_le; Er_su) among root (white bars), stem (gray bars) and branch xylem (black bars).** Values are means ± SE.

The empirically determined sapwood area-specific hydraulic conductivity (*K*_S_^emp^) of root segments differed by a factor of 100 across species with *E. subumbrans* showing the highest values by far, whereas the smallest values were observed in roots of *T. cacao* (**Figure 2**; Table A2). The differences in *K*_S_^emp^ of branches across species were much less pronounced (2.3 to 7.4 kg m^-1^ MPa^-1^ s^-1^) with *G. gnemon* showing the highest and *T. cacao* the lowest numbers. Overall, root segments always showed higher hydraulic conductivities than branches (‘lme’; *p* < 0.001). Furthermore, *K*_S_^emp^ values (in both root and branch segments) were always smaller than the theoretically calculated hydraulic conductivity (*K*_S_^theo^) as derived from vessel diameters by Hagen–Poiseuille’s law. Even though branch and root segments around 30 cm lengths were used, probably open-cut vessels could not be avoided particularly for root segments of *E. subumbrans*. However, mean *K*_S_^emp^ values reached 9–45% of respective* K*_S_^theo^ values indicating that open-cut vessels were negligible for most species, except for *G. gnemon* where 50–81% of respective* K*_S_^theo^ values were measured (**Figure 2**). Empirically measured and calculated specific conductivity in root segments showed a positive linear relationship (‘lme’; *p* < 0.001), but not for branch segments (‘lme’; *p* = 0.71).

### Anatomical Differences across Species in Root, Stem, and Branch Wood Properties

**FIGURE 3:**
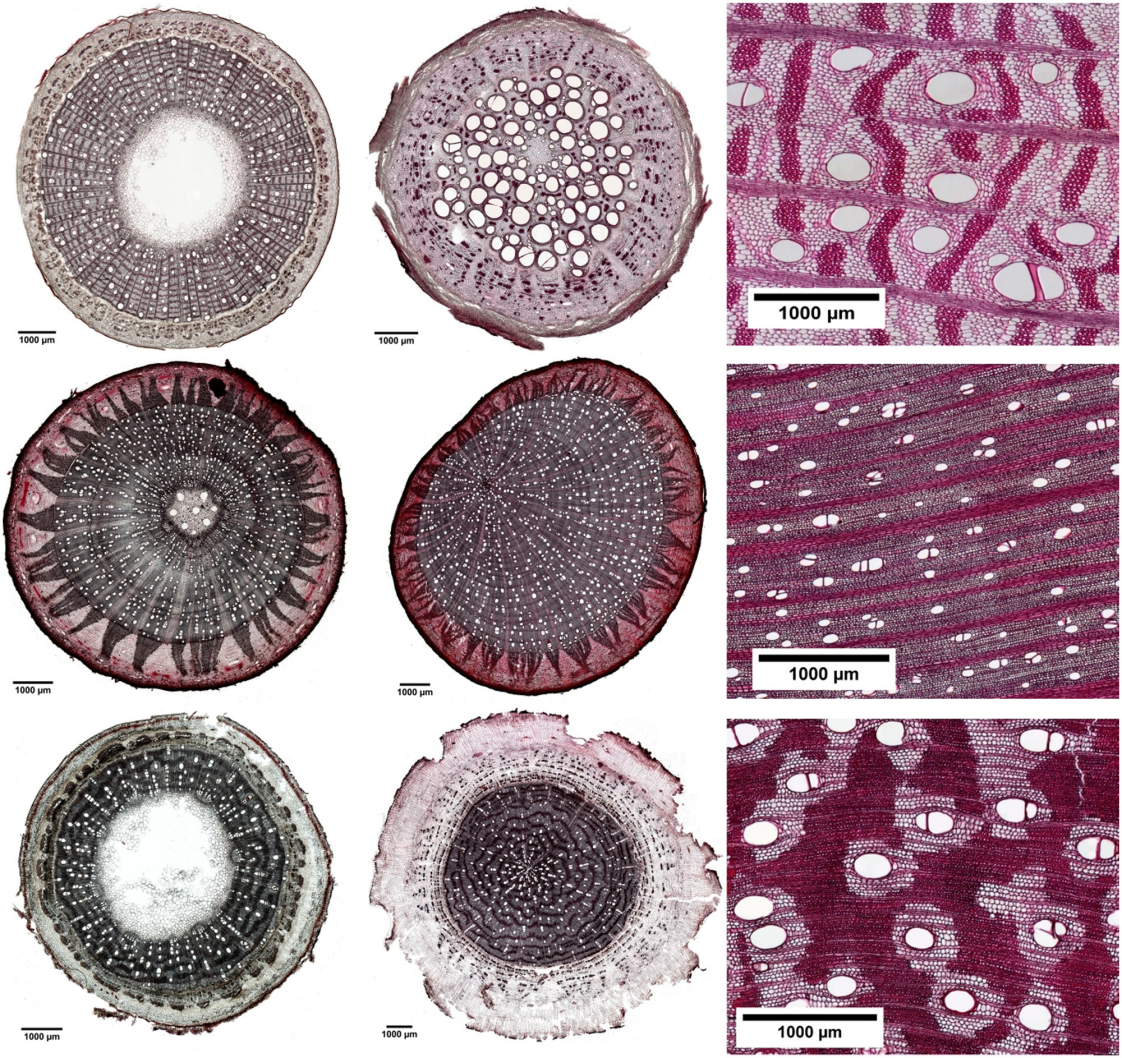
**Cross-sections of different tree parts along the flow path: branch **(left row)**, roots **(middle row)**, and stems **(right row)** for three common tree species from cocoa agroforests in Sulawesi, Indonesia. *Erythrina subumbrans***(upper line)**, *Theobroma cacao***(middle line),** and *Gliricidia sepium***(lower line)**.** The scale bars are presented in the figures and black bars represent 1000 μm.

**FIGURE 4:**
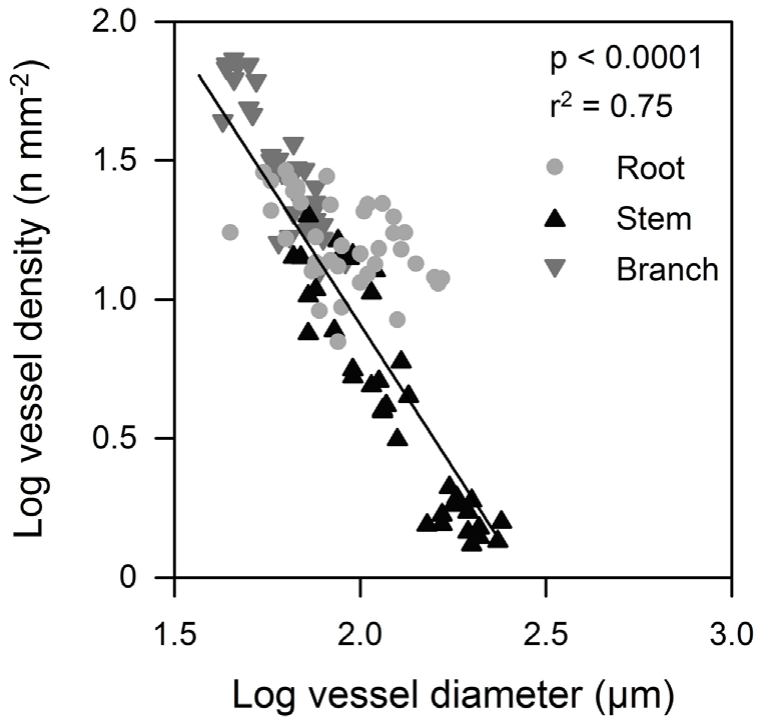
**Mean vessel diameter in relation to vessel density in tree organs (roots, stems, and branches) along the flow path for the six tree species**.

We found considerable variation in wood anatomical and derived hydraulic traits along the flow path from root, to stem and branch wood for all six species. Exemplary pictures for this variation from three of the species are given in **Figure 3**. In four of the six species average vessel diameter (*d*) was significantly largest in the stem and not in the root wood; in the remaining two species *d* was comparable between root and stem wood (**Figure 2**). Along the flow path smallest vessels were always observed in the branch wood of all species with the exception of *D. zibethinus* (**Figure 2**). The same pattern was observed for the hydraulically weighted vessel diameter (*d*_h_) for branch wood, while the differences in *d*_h_ between root and stem wood were only significant in *T. cacao* and *D. zibethinus*. In general, several wood anatomical and derived hydraulic traits allowed a grouping between the three perhumid tree species originating from strictly wet tropical environments, and the three seasonal tree species reported to tolerate moderate droughts. As mentioned above, *d*_h_ was not significantly higher in stem than in root wood for the three seasonal tree species, and VD was comparable between root and branch wood and did not differ significantly. On the other hand, highest vessel densities were observed in the branch wood of all perhumid tree species, although differences were only significant in two of the three species. However, when comparing the two groups (perhumid vs. seasonal) significant differences were found (‘lme’; *p* < 0.001). In general, VD varied considerably between the organs and species as well and was found to decrease in the order branch – root – stem across all six species (**Figure 2**). Variation in VD numbers was lowest (factor < 2) in the root xylem and highest (factor > 10) in the stem xylem. VD decreased exponentially with increasing vessels diameter; we therefore concentrate on changes in *d* along the flow path in the following (**Figure 4**).

Relative vessel lumen area (*A*_lumen_), i.e., the ratio of lumen to sapwood area, was lowest in the stem wood in all species except of *G. gnemon* (**Figure 2**). Root and branch wood showed therefore higher *A*_lumen_ values that tended to show similar dimensions with the exception of the seasonal tree species that showed significantly higher *A*_lumen_ values in the root segments.

When concentrating on hydraulic properties we found a similar pattern in *K*_S_^theo^, where hydraulic conductivity was highest in roots of seasonal tree species (‘lme’; *p* < 0.001), while there is no overall significant difference between the root and stem wood in the perhumid species (*p* > 0.05).

### Leaf Morphological, Chemical, and Hydrological Properties

Specific leaf area of sun-exposed leaves was higher by roughly a factor of two in *E. subumbrans*, *G. sepium*, and* L. leucocephala* than in *D. zibethinus*, *T. cacao*, and *G. gnemon* (**Table 3**). The sapwood to leaf area ratio (‘Huber value,’ HV) of the sun-lit branch samples was lowest in *G. sepium* and *T. cacao*, and highest in *E. subumbrans*. Empirical leaf area-specific hydraulic conductivity (*K*_L_^emp^) in these branch samples showed a large variation across species ranging from 1.2 10^-4^ kg m^-1^ MPa^-1^ s^-1^ in *T. cacao* over 4.3–4.8 10^-4^ kg m^-1^ MPa^-1^ s^-1^ in *D. zibethinus*, *G. sepium*, *E. subumbrans*, and *L. leucocephala* to 14.2 10^-4^ kg m^-1^ MPa^-1^ s^-1^ in *G. gnemon*. The variation in theoretical leaf area-specific hydraulic conductivity (*K*_L_^theo^) derived from the wood anatomical properties was less pronounced. Lowest values were found in *T. cacao*, followed by *G. sepium*, while the other four tree species had ca. 2–5 times higher *K*_L_^theo^ values (**Table 3**).

**Table 3:** Leaf morphological, hydraulic, and chemical properties of the six investigated tree species.

Species	SLA (cm^2^ g^-1^)	HV (m^2^ m^-2^)	K_L_^emp^ × 10^-4^ (kg m^-1^ MPa^-1^ s^-1^)	K_L_^theo^ × 10^-4^ (kg m^-1^ MPa^-1^ s^-1^)	N_leaf_ (g kg^-1^)	δ^13^C (‰)
**Perhumid**
* T. cacao*	125.76 ± 8.38 a 3 (9)	1.34 ± 0.33 a 6	1.23 ± 0.75 a 2	9.90 ± 1.89 a 6	1.87 ± 0.25 a 6 (18)	–29.45 ± 0.39 ab 6 (18)
*D. zibethinus*	124.74 ± 12.19 a 3 (9)	3.07 ± 0.99 a 6	4.27 ± 0.92 a 6	51.11 ± 13.18 b 6	2.28 ± 0.25 a 6 (18)	–29.87 ± 0.46 a 6 (18)
*G. gnemon*	146.75 ± 5.35 a 3 (9)	2.90 ± 0.71 a 6	14.23 ± 4.22 b 6	29.81 ± 10.57 ab 6	2.69 ± 0.21 ab 6 (18)	–29.83 ± 0.42 a 6 (18)
**Seasonal**
* G. sepium*	271.48 ± 19.74 b 3 (9)	1.72 ± 0.26 a 6	4.29 ± 1.09 a 6	13.31 ± 1.97 ab 6	3.35 ± 0.17 be 6 (18)	–29.09 ± 0.19 a 6 (18)
*L. leucocephala*	293.2 ± 21.3 b 3 (6)	2.07 ± 0.52 a 6	4.79 ± 0.81 a 6	47.16 ± 11.23 ab 6	3.59 ± 0.12 c 6 (18)	–27.93 ± 0.08 c 6 (18)
*E. subumbrans*	264.38 ± 11.11 b 3 (9)	3.91 ± 1.40 a 6	4.76 ± 1.69 a 6	38.25 ± 14.05 ab 6	3.59 ± 0.14 c 6 (18)	–27.89 ± 0.28 be 6 (18)

Values are means ± SE; the number of investigated trees and measured samples (in parentheses) is also given. Different small letters indicate differences between species. See **Table 1** for definition of abbreviations.

Mass-specific foliar nitrogen concentration (*N*_leaf_) was lowest in *T. cacao*, medium high in *D. zibethinus* and *G. gnemon*, and highest in the three seasonal species *G. sepium*, *L. leucocephala*, and *E. subumbrans* (**Table 3**). Variation in leaf carbon isotopic composition was rather small. The two species *E. subumbrans* and *L. leucocephala* revealed a ca. 1.0–1.8 higher δ^13^C value than the four other species that did not show significant differences in this variable.

### Interrelationships between Vascular Properties, Tree Stem Growth, and Hydraulic Conductivity

**FIGURE 5:**
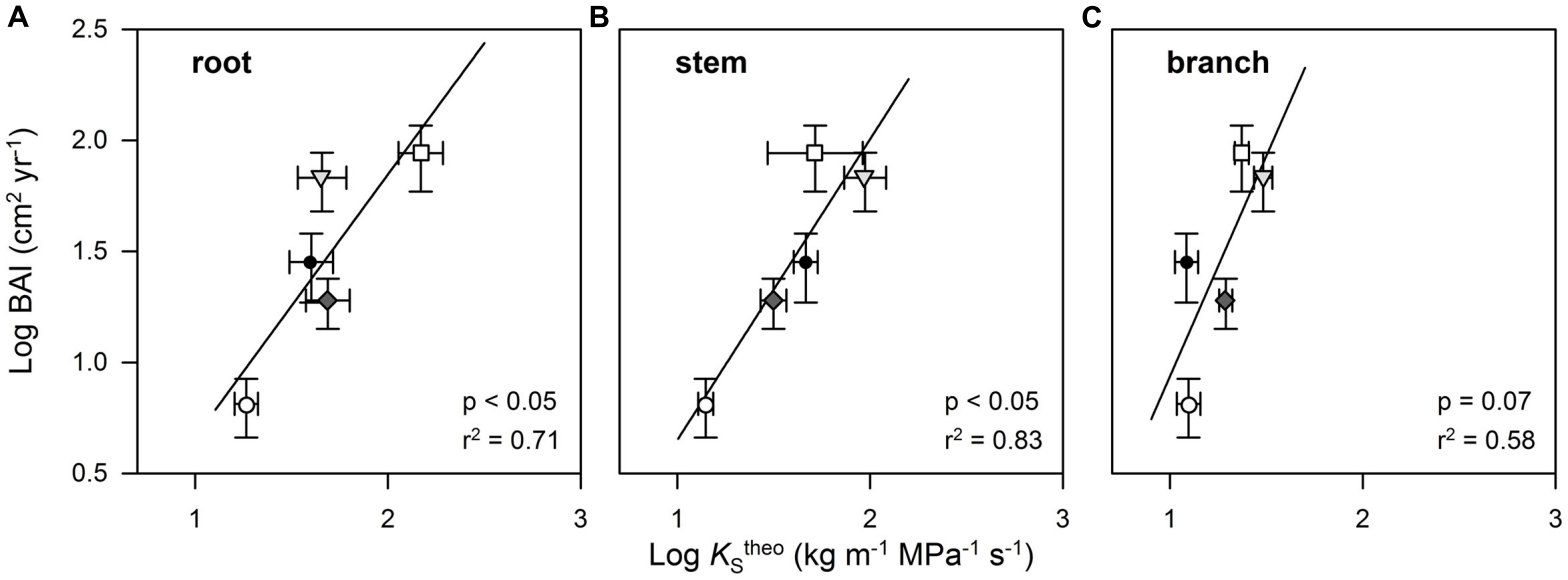
**Relationship between stem basal area increment (BAI) of cacao and four shade tree species and theoretically calculated cross sectional sapwood area-specific hydraulic conductivity (*K*_**S**_^**theo**^) in the root **(A)**, stem **(B)**, and branch wood **(C)**.** Each symbol represents mean values for each tree species (○ Th_ca; ∇ Du_zi; ⧫ Gl_se; □ Le_le; ● Gn_gn). Error bars indicate 1 SE.

A PCA on the inter-relationships between the investigated traits explained a large proportion of the total variance of the data set along the first four axes (**Table 4**). The first axis was strongly positively associated with all wood anatomical traits (including HV and δ^13^C), but negatively with WD. Axis 1 was furthermore positively related to DBH. Axis 2 was strongly associated with stem and branch lumen area as well as with the leaf traits (*K*_L_^emp^ and *N*_leaf_). In contrast to *K*_S_^emp^ in root segments, branch *K*_S_^emp^ was associated with axis 2 and therefore showed an inter-relationship with *K*_L_^emp^. BAI showed an only moderate association with the first axis and thus was only weakly correlated with the majority of wood anatomically and tree structural variables. BAI was correlated best with the third axis that was only associated with the variables AGB, DBH, and WD (positively), as well as root *K*_S_^theo^ and *K*_S_^emp^ (negatively). A Pearson’s coefficient of correlation analysis, however, revealed a strong relationship between BAI and *K*_S_^theo^ on a species level for root, stem, and branch wood tissue (**Figures 5A–C**).

**Table 4:** Results of a Principal Components Analysis (PCA) on the response of six agroforestry tree species with respect to stem BAI, anatomical properties of the coarse root, stem and branch wood as well as hydraulic and leaf traits.

	Axis 1 (EV 0.46)		Axis 2 (EV 0.21)		Axis 3 (EV 0.16)		Axis 4 (EV 0.12)	
AGB	0.31	(0.10)	–0.25	(0.16)	**0.87**	(0.93)	0.06	(0.93)
DBH	**0.81**	(0.65)	0.08	(0.65)	0.54	(0.94)	0.06	(0.94)
BAI	0.17	(0.03)	–0.09	(0.04)	**0.83**	(0.72)	0.52	(1.00)
WD	–0.58	(0.34)	–0.05	(0.34)	**0.75**	(0.90)	–0.11	(0.94)
*A*_lumenroot_	**0.90**	(0.81)	–0.03	(0.81)	–0.03	(0.81)	–0.42	(0.99)
*A*_lumenstem_	–0.29	(0.08)	**0.79**	(0.70)	0.43	(0.88)	–0.32	(0.98)
*A*_lumenbranch_	–0.12	(0.01)	**0.93**	(0.88)	–0.11	(0.90)	0.21	(0.94)
*d*_hroot_	**0.92**	(0.84)	0.13	(0.86)	–0.11	(0.87)	–0.36	(1.00)
*d*_hstem_	**0.91**	(0.82)	0.11	(0.83)	–0.17	(0.86)	0.32	(0.97)
*d*_hbranch_	**0.82**	(0.68)	–0.37	(0.81)	0.04	(0.82)	0.27	(0.89)
*K*_s_^theo^ root	**0.88**	(0.77)	0.10	(0.78)	–0.26	(0.84)	–0.39	(1.00)
*K*_s_^theo^ stem	**0.88**	(0.78)	0.36	(0.91)	–0.05	(0.92)	0.27	(0.99)
*K*_s_^theo^ branch	**0.89**	(0.79)	–0.09	(0.80)	0.06	(0.80)	0.39	(0.95)
*K*_s_^emp^ root	**0.85**	(0.72)	0.18	(0.76)	–0.18	(0.79)	–0.45	(0.99)
*K*_s_^emp^ branch	–0.29	(0.08)	**0.66**	(0.52)	0.31	(0.62)	–0.40	(0.78)
*K*_s_^emp^	–0.21	(0.04)	**0.87**	(0.79)	0.35	(0.92)	–0.27	(0.99)
*K*_s_^theo^	**0.78**	(0.61)	0.39	(0.76)	0.26	(0.83)	0.41	(1.00)
HV	**0.77**	(0.59)	0.64	(0.51)	–0.02	(0.70)	0.05	(0.98)
*N*_leaf_	–0.23	(0.05)	**0.68**	(0.51)	–0.44	(0.70)	0.53	(0.98)
δ^13^*C*	**0.71**	(0.50)	–0.41	(0.67)	0.28	(0.74)	–0.49	(0.99)

Given are the loadings of the selected variables along the four main explanatory axes as well as the cumulative *r*^2^ values (in brackets) for a given variable. Numbers below the four axes indicate the eigen values (EV) of the axes. Numbers in bold indicate the variables with the closest relation to the respective axis.

A systematic correlation analysis of pairs of traits confirmed that most of the inter-relationships identified in the PCA on a species level were also valid on a tree individual level (**Table 5**). BAI was strongly interrelated with AGB as well as DBH and showed moreover a significant correlation with stem *d*_h_ and stem *K*_S_^theo^. None of these three variables were correlated with WD that generally showed only few and moreover relatively low correlations with other variables in the Pearson correlation analysis. Mixed effect models incorporating the pseudoreplication due to species confirmed that WD does not explain neither *K*_S_^theo^, *d*_h_ nor BAI in our data well (‘lme’; *p* > 0.05).

**Table 5:** Pearson’s coefficients of correlation between pairs of traits.

	BAI	AGB	DBH	WD	*A*_lumen_ _root_	*A*_lumen_ _stem_	*A*_lumen_ _branch_	*d*_h_ _root_	*d*_h_ _stem_	*d*_h_ _branch_	*K*_s_^theo^ _root_	*K*_S_^theo^ _stem_	*K*_S_^theo^ _branch_	*K*_S_^emp^ _branch_	*K*_S_^emp^ _root_	*K*_L_^emp^	*K*_L_^theo^	HV	δ^13^C
AGB	**0.77**																		
DBH	**0.82**	**0.93**																	
WD	n.s.	n.s.	n.s.																
*A*_ lumen root_	n.s.	n.s.	0.45	–0.41															
*A*_ lumen stem_	n.s.	0.38	n.s.	0.37	n.s.														
*A*_ lumen branch_	n.s.	n.s.	n.s.	n.s.	n.s.	0.36													
*d*_ h root_	n.s.	0.34	0.47	–0.38	**0.83**	n.s.	n.s.												
*d*_ h stem_	0.63	0.50	**0.64**	–0.52	0.55	n.s.	n.s.	**0.68**											
*d*_ h branch_	n.s.	n.s.	n.s.	n.s.	0.45	n.s.	n.s.	0.52	**0.80**										
*K*_s_^theo^_root_	n.s.	0.35	0.49	–0.40	**0.94**	n.s.	n.s.	**0.97**	**0.64**	0.50									
*K*_s_^theo^_stem_	**0.70**	**0.68**	**0.73**	n.s.	0.48	0.36	n.s.	**0.64**	**0.87**	**0.59**	**0.59**								
*K*_s_^theo^_branch_	0.48	n.s.	0.35	n.s.	0.40	n.s.	n.s.	0.44	**0.79**	**0.86**	0.44	**0.66**							
*K*_s_^emp^_branch_	n.s.	n.s.	n.s.	n.s.	n.s.	n.s.	n.s.	n.s.	n.s.	n.s.	n.s.	n.s.	n.s.						
*K*_s_^emp^_root_	n.s.	n.s.	0.42	n.s.	**0.66**	0.36	n.s.	**0.74**	n.s.	n.s.	**0.75**	0.46	n.s.	n.s.					
*K*_L_^emp^	n.s.	n.s.	n.s.	n.s.	n.s.	0.43	n.s.	n.s.	n.s.	n.s.	n.s.	n.s.	n.s.	**0.75**	n.s.				
*K*_L_^theo^	n.s.	0.44	0.44	n.s.	0.35	n.s.	n.s.	0.42	**0.58**	0.54	0.40	**0.66**	**0.64**	n.s.	n.s.	n.s.			
HV	n.s.	n.s.	n.s.	n.s.	n.s.	0.40	n.s.	n.s.	n.s.	n.s.	n.s.	0.35	n.s.	n.s.	n.s.	0.43	**0.82**		
δ^13^C	n.s.	n.s.	n.s.	n.s.	**0.58**	n.s.	n.s.	0.38	n.s.	n.s.	0.49	n.s.	n.s.	n.s.	n.s.	n.s.	n.s.	n.s.	
*N*_ leaf_	n.s.	n.s.	n.s.	n.s.	n.s.	n.s.	0.54	n.s.	n.s.	n.s.	n.s.	n.s.	n.s.	n.s.	n.s.	n.s.	n.s.	n.s.	–0.40

Highly significant correlations are shown in bold (*p* < 0.001), non-significant correlations (*p* > 0.05) are n.s. The correlation analysis was based on comparisons on tree individual level.

Contrary to significant relationships of stem wood *K*_S_^theo^ with AGB, BAI and DBH, no relationship of *K*_S_^emp^ between any of these traits could be found, except for *K*_S_^emp^ in root segments that were related to DBH.

As expected, all species and organs showed a positive relationship between *K*_S_^theo^ and *d*_h_ (**Table 5**). Foliar nitrogen content (*N*_leaf_) as well as the carbon isotope signature (δ^13^C) did not show any relation with neither leaf area-specific hydraulic conductivity (*K*_L_^theo^), *K*_S_^emp^ nor HV, but a strong significant correlation within each other. *N*_leaf_ was unrelated to BAI among species (*p* > 0.1, *r* = 0.05) also when excluding the three seasonal species.

Stepwise model selection confirmed that *K*_S_^theo^ is the best predictor for AGB together with WD and neither *N*_leaf_, δ^13^C nor *K*_S_^emp^ were explaining the variability in our data significantly.

## Discussion

### Patterns in Xylem Anatomy among Species in Stem, Root, and Branch Wood

Most studies on hydraulic anatomical properties in trees describe vessel sizes to be largest in roots and basipetally taper to the branches (Tyree and Zimmermann, 2002; McElrone et al., 2004; Goncalves et al., 2007; Domec et al., 2009; Lintunen and Kalliokoski, 2010). The first notification of this paradigm even goes back to observations by Nehemiah Grew in the 17th century (Baas, 1982). Generally, variation in conduit diameter is a compromise between hydraulic efficiency, safety, and the maximization of conductivity per growth investment due to conduit tapering (Sperry et al., 2008, 2012). As concluded by Tyree and Zimmermann (2002) the reason for conduit tapering is the control of water distribution, and more importantly to counter increases in flow resistance and gravimetrically forces with tree height to minimize the increasing risk of xylem dysfunction with path length (West et al., 1999; Anfodillo et al., 2006; Sperry et al., 2012). This is additionally mirrored in the meanwhile commonly observed relation between conduit size and vulnerability to cavitation (Wheeler et al., 2005; Maherali et al., 2006; Cai et al., 2010; Domec et al., 2010; Sterck et al., 2012). It is argued that in roots water stress will not be as great as in stems, since water potentials drop in going from root to stem to leaf (Tyree and Zimmermann, 2002). As long as soil water is still extractable, roots would then be less exposed to drought-induced embolism and might therefore afford larger vessels. Furthermore, with a small need of structural support and embedded in a soil matrix, biomechanical stress is unlikely to affect roots (McElrone et al., 2004; Pratt et al., 2007). Additionally, most plants have developed a mechanism to restore vessel functionality by refilling embolized vessels through living rays and paratracheal parenchyma. The contribution of paratracheal parenchyma was recently shown for grapevine by Brodersen et al. (2010), while the molecular and physiological paths were investigated by Chitarra et al. (2014). As coarse roots are located close to the water source it seems beneficial to restrict hydraulic failure to areas within the complex hydraulic network that are easily refilled. Embolism reversal is thought to occur by active transport of sugars into empty conduits, which are generally accumulated in high amounts within a trees rooting system (Würth et al., 2005).

Our results are in contrast to the common assumption as we found the largest vessels along the flow path in the stem xylem and not in the roots. Supporting our first hypothesis, our results are in accordance with the observations of a hump-shaped vessel size distribution along the flow path found in tropical trees of Indonesia (Schuldt et al., 2013), supported by findings from South America where the largest vessels were observed in the stem wood (Machado et al., 2007) or comparing just root and branch wood both organs showed similar vessel sizes (Fortunel et al., 2013). Our confirmative finding could represent a response to permanent water availability and low evaporative demand in this humid region, where trees without severe drought limitation might have developed roots with large relative lumen area and less structural tissue that can achieve sufficiently high axial conductivities in these organs. Thereby they would compensate for the smaller vessel diameters in roots than in the trunk in accordance with the pipe model theory by Shinozaki et al. (1964). Originally, this theory attempted to explain plant architecture in a quantitative way by proposing that photosynthetic organs should be supported by an adequate structure of non-photosynthetic organs in order to guarantee functionality (Chiba, 1998). Nevertheless, one has to keep in mind that the pipe model theory is not a hydraulic model, but should be viewed as a biomass allometry model with no particular implications concerning either hydraulics or biomechanics as proposed by McCulloh and Sperry (2005).

Machado et al. (2007) argued that shallow roots of moist tropical forest species, which is a common rooting pattern in tropical moist forests (Leuschner et al., 2006; Hertel et al., 2009), are subjected to variations in water availability and the narrower vessels in the root wood are a safety trade-off against cavitation. However, the vessel sizes found in coarse roots in the present study as well as in other tropical studies (Machado et al., 2007; Fortunel et al., 2013; Schuldt et al., 2013) are at least 30% larger compared to, e.g., temperate forest tree species (Köcher et al., 2012), and therefore might not directly be rated as an increased safety against cavitation compared to the stem or branch wood. It seems rational to assume that not the coarse root xylem, despite large vessel sizes, represents the most drought-sensitive organ, but rather that of fine roots with smaller diameter. In contrast to coarse roots, which are primarily responsible for axial water transport, fine roots represent the highest resistance for water transport within the rooting system due to radial water flow either along the apoplastic or cellular pathway (Steudle, 2000). As the most distal organs they are scarified in response to drought in order to avoid serious harm to coarse and large roots like it has been observed in various temperate and boreal forests (Gaul et al., 2008; Chenlemuge et al., 2013; Hertel et al., 2013). Fine roots might thereby act as a sort of ‘hydraulic fuse,’ which evolved from Zimmermann’s segmentation hypothesis (Tyree and Zimmermann, 2002) in analogy to the leaf petiole (Zufferey et al., 2011). At the root level this ‘hydraulic segmentation’ might additionally protect the below-ground system preventing the reverse water flow from main to lateral roots and back to the dryer soil as discussed for grapevine by Lovisolo et al. (2008). Woody plants would accordingly restrict hydraulic failure to redundant organs that are readily replaced (Sperry et al., 2002) although it has been argued that the term ‘hydraulic fuse’ should be reconsidered since roots are not necessarily an ‘expandable’ organ (Gonzalez-Benecke et al., 2010). The construction costs of fine roots and lignified small-diameter roots in term of carbon and nutrients may not be much smaller than for twigs and leaves, and the loss of roots is directly related to the loss of absorption capacity for nutrients and water. However, comparable data on fine root mortality and percentage loss of conductivity in coarse roots that would empirically support the idea that fine root are sacrificed in order to protect the hydraulic system are to our knowledge not available so far.

Concurrently with decreasing vessel size, conduit frequency is generally reported to increase from the roots to the branches (e.g., Lintunen and Kalliokoski, 2010). This commonly observed trade-off between VD and vessel diameter (Preston et al., 2006; Sperry et al., 2008; Zanne et al., 2010) could not be confirmed in our study where the stem wood showed by far the lowest VD compared to roots and branches. Since flow in capillary systems is proportional to the fourth power of vessel radius according to Hagen–Poiseuille law, variations in vessel diameter has a much greater effect on *K*_S_^theo^ than variations in VD. However, due to the occurrence of a higher relative vessel lumen area and a few large vessels in roots we have observed that specific conductivity in the three seasonal species, i.e., *G. sepium, E. subumbrans*,* and L. leucocephala*, was higher in roots than in stems, even though the largest vessels were observed in the stem wood. At least for *E. subumbrans and L. leucocephala* this pattern could additionally be explained by the highest relative vessel lumen area found in roots, i.e., less space was occupied by tracheids or fibers compared to the stem or branch wood. Furthermore, disproportionately high empirical conductance measured in *E. subumbrans* might be attributed to the presence of open-cut vessels, which are highly conductive as water does not have to pass pit membranes, which generally account for more than 50% of the total hydraulic resistance (Choat et al., 2008). However, since tree hydraulic traits have been associated with general habitat preferences of various species (Sperry, 2000; Maherali et al., 2004), this finding could be due to the biogeographic background and could represent genetically determined adaptations to different water availability in the natural habitat of the species. While *T. cacao,* *D. zibethinus* and* G. gnemon* are known to be strictly wet tropical forest trees rather sensitive to drought and low air humidity (Brown, 1997; Carr and Lockwood, 2011), the other three species are reported to be fast-growing drought-resistant trees (Mrema et al., 1997; Fagbola et al., 2001). While drought resistance is recorded for some *Erythrina* species (da Silva et al., 2010; Manoharan et al., 2010), not many data are available on *E. subumbrans*, which is a species native to Indonesia. In a habitat where water stress is generally modest or absent such as the humid climate in Sulawesi, cavitation-avoiding mechanisms might be less beneficial than hydraulic efficiency and largest vessels can occur in stem xylem, thereby reducing the hydraulic resistance along the flow path. However, it remains speculative why the largest vessels along the flow path are observed in the root xylem only in biomes that frequently experience either drought- or frost stress. The size of a vessel is thought to be caused by the concentration of the plant hormone indole-3-acetic acid (IAA), an endogenous auxin, at the time of cell differentiation (Aloni, 1987; Lovisolo et al., 2002), which is also related to the cambial age and related cambial activity as seen by the radial increase in vessel size at the stem base of a tree (Spicer and Gartner, 2001). It would thus be of interest to extent the results of the present study to a quantification of IAA concentration in both coarse root and stem cambium in tropical and temperate trees; the latter should show higher concentrations in the root xylem independently of cambial age in agreement with the common paradigm that largest vessels are found in the rooting system.

### Relationships between Vascular Properties, Tree Stem Growth, and Hydraulic Conductivity

Wood density is an easy to measure functional wood property that has been linked to various ecological and other functional traits. In species showing a relatively large fraction of vessels close to the hydraulically weighted mean vessel diameter (*d*_h_), *K*_S_^theo^ should correlate negatively with WD (Bucci et al., 2004; Meinzer et al., 2008; Gonzalez-Benecke et al., 2010). Similar to observations on tropical forest trees from perhumid tropical environments (Poorter et al., 2010; Schuldt et al., 2013) we expected WD to be unrelated to wood anatomical and hydraulic properties. Even though we found a correlation between WD and *d*_h_ on tree level, this relationship could not be confirmed on species level accounting for species pseudoreplication in mixed effect models. Also we found no significant relationship of WD to basal stem area increment, contradicting former results on a close relation between WD and growth for tropical trees (King et al., 2006; Poorter et al., 2010; Hietz et al., 2013). Several other studies report WD to be partially decoupled from hydraulic conductivity due to variation of frequency and size of fibers in angiosperms (Preston et al., 2006; Martinez-Cabrera et al., 2009; Zanne et al., 2010). Results on the relationship between WD and vascular properties as well as tree growth are thus partly conflicting; while some studies confirm that WD varies inversely with vessel size (Preston et al., 2006; Jacobsen et al., 2007; Thomas et al., 2007; McCulloh et al., 2011; Gleason et al., 2012), others did not support this finding (Martinez-Cabrera et al., 2009; Poorter et al., 2010; Russo et al., 2010; Zanne et al., 2010; Fan et al., 2012). These contradicting results are indicating that the relation between WD and growth or vessel traits is not necessarily interrelated and should be viewed separately. We further suspect that the relation between wood properties and tree hydraulics may depend as well on biogeographical origin and drought-adaptation strategy of the species investigated since convergent environmental factors such as water availability are known to lead to adaptations in functional wood anatomical properties (Swenson and Enquist, 2007; Gleason et al., 2013; Richardson et al., 2013).

We found wood anatomical and derived hydraulic properties to be a much better predictor for tree stem growth performance than WD as *K*_S_^theo^ of the all tree organs studied were strongly positively correlated with stem BAI on a species level. This is in accordance with a growing body of studies showing strong links between growth rate and wood anatomical traits (Zhang and Cao, 2009; Poorter et al., 2010; Russo et al., 2010; Fan et al., 2012). In contrast, neither empirically measured branch and root *K*_S_^emp^, nor foliar δ^13^C or foliar nitrogen content were good predictors for aboveground growth performance.

We expected to find close correlations between functional leaf traits assumed to be associated with high aboveground productivity, i.e., high foliar N content and more negative foliar δ^13^C, and stem increment in our samples. However, no such correlation was found. This is most likely explained by the fact that our sampled species contained several N-fixing legume species, our relatively low species number as well as due to the fact that our study was conducted in a perhumid region were drought stress is not to be expected.

## Conclusion

Our study results suggest that even though vessel traits, growth performance, and WD relations follow distinct conceptually determined trade-offs, some of these long-established paradigms might not be uniformly applicable to tree species from all biogeographic regions presumably due to their varying drought adaptation strategies. In moist tropical environments we could not confirm the paradigm of continuous conduit tapering from roots to branches although some traits (VD, relative vessel lumen area, and theoretical sapwood area-specific conductivity) enabled a clear separation between the three strictly wet tropical species and the three seasonal tree species. We therefore expect patterns in vessel traits along the flow path from roots to branches to be dependent on the long-term precipitation regime at the biogeographic origin of the investigated tree species. Furthermore and contrary to common knowledge, the investigated tree species did neither show a relationship between aboveground growth performance and WD nor foliar nitrogen content, nor between WD and vessel size. Instead, we found growth rate to be closely linked with wood anatomical and derived hydraulic traits. Future research should thus include a systematic approach to different biogeographic regions and cover a wider range of ecosystem types particularly underrepresented biomes.

## Conflict of Interest Statement

The authors declare that the research was conducted in the absence of any commercial or financial relationships that could be construed as a potential conflict of interest.

## Footnotes

1 http://rsb.info.nih.gov/ij
