# Supplementary material for: Patterns in hydraulic architecture from roots to branches in six tropical tree species from cacao agroforestry and their relation to wood density and stem growth
Source: Front Plant Sci. 2015 Mar 31;6:191. doi: 10.3389/fpls.2015.00191 (PMC4379754; doi:10.3389/fpls.2015.00191)
Supplement: Supplementary file 1 [file data_sheet_1.pdf]

Supplementary Material

Table A1: Results from linear regression analyses between branch and root cross-sectional area (A_{cross}, mm^2) and corresponding xylem cross-sectional area (A_{xylem}, mm^2) without pith and bark for the eight tree species, and averaged across species for branches and roots. Given are sample number, intercept of the x- and y-axis, slope, p-value, coefficient of determination and the xylem to cross-sectional area ratio (A_{xylem} / A_{cross}, mean ± SE).

Species	Organ	n	$A_{xylem} = a + b \times A_{cross}$					A_{xylem} / A_{cross}
			y(0)	a	b	p	r^2	
Th_ka	branch	6	4.03	-2.0914	0.5196	<0.001	0.98	0.48 ± 0.01
Er_su	branch	6	12.61	-7.4852	0.5936	0.021	0.68	0.45 ± 0.04
Du_zi	branch	6	27.53	-18.8424	0.6844	0.001	0.94	0.49 ± 0.02
Gl_se	branch	6	7.42	-4.7291	0.6375	0.002	0.92	0.56 ± 0.04
Le_le	branch	6	-0.67	0.4622	0.6875	<0.001	0.99	0.70 ± 0.01
Gn_gn	branch	6	-2.54	1.6189	0.6378	0.001	0.96	0.66 ± 0.01
All	*branch*	*36*	*-0.91*	*0.5033*	*0.5507*	*<0.001*	*0.76*	*0.56 ± 0.02*
Th_ka	root	6	0.14	-0.0863	0.6161	<0.001	0.99	0.61 ± 0.01
Er_su	root	8	5.82	-2.6486	0.4547	<0.001	0.89	0.40 ± 0.02
Du_zi	root	8	3.25	-2.2785	0.7020	<0.001	0.96	0.64 ± 0.01
Gl_se	root	5	23.40	-13.1864	0.5636	0.016	0.72	0.42 ± 0.05
Le_le	root	6	-13.21	4.6253	0.3501	0.006	0.84	0.43 ± 0.02
Gn_gn	root	6	519.48	58.8047	-0.1132	0.225	0.15	0.25 ± 0.05
All [1)]	*root*	*33*	*-0.84*	*0.4062*	*0.4830*	*<0.001*	*0.68*	*0.51 ± 0.02*

[1)] Gn_Gn excluded from regression analysis.

Table A2: Wood anatomical and hydraulic properties of the root, stem and branch wood of the six studied agroforestry tree species. Given are means ± SE and the number of investigated trees and measured samples (in parentheses). Lowercase letters indicate statistically significant differences between species within same organ and letters in parentheses indicate significant differences between organs within a given species. See Table 1 for definition of abbreviations.

	K_S^{emp} (kg m^{-1} MPa^{-1} s^{-1})	K_S^{theo} (kg m^{-1} MPa^{-1} s^{-1})	d (µm)	d_h (µm)	VD (n mm^{-2})	A_{lumen} (%)
Perhumid						
Theobroma cacao						
root	2.64 ± 0.3 a (a) 6 (6)	18.4 ± 2.56 a (a) 6 (6)	63.63 ± 2.01 a (b) 6 (6)	86.71 ± 3.22 a (a) 6 (6)	25.19 ± 1.11 b (b) 6 (6)	9.20 ± 0.76 a (b) 6 (6)
stem		14.03 ± 1.26 a (a) 6 (6)	72.88 ± 2.29 a (c) 6 (6)	112.18 ± 5.04 a (b) 6 (6)	11.04 ± 1.23 c (a) 6 (6)	5.29 ± 0.35 a (a) 6 (6)
branch	2.33 ± 0.94 a (a) 2 (4)	12.48 ± 1.74 a (a) 6 (6)	51.43 ± 2.77 a (a) 6 (6)	71.28 ± 5.88 a (a) 6 (6)	44.42 ± 6.39 bc (c) 6 (6)	9.95 ± 0.92 ab (b) 6 (6)
Durio zibethinus						
root	5.99 ± 4.8 a (a) 6 (8)	45.45 ± 12.87 ab (a) 6 (8)	79.13 ± 7.64 ab (a) 6 (8)	129.01 ± 15.35 ab (a) 6 (8)	18.01 ± 3.23 ab (b) 6 (8)	8.88 ± 1.12 a (ab) 6 (8)
stem		94.11 ± 23.14 bc (a) 6 (6)	189.81 ± 13.8 d (b) 6 (6)	239.45 ± 16.86 c (b) 6 (6)	1.66 ± 0.11 a (a) 6 (6)	5.46 ± 0.74 a (a) 6 (6)
branch	3.49 ± 0.61 a (a) 5 (5)	30.39 ± 3.32 b (a) 6 (6)	69.00 ± 2.98 bc (a) 6 (6)	105.19 ± 6.23 b (a) 6 (6)	25.95 ± 2.8 ab (b) 6 (6)	10.26 ± 0.59 ab (b) 6 (6)
Gnetum gnemon						
root	39.14 ± 14.45 a (a) 6 (7)	40.03 ± 10.18 ab (ab) 6 (6)	89.4 ± 5.73 b (b) 6 (6)	122.93 ± 7.39 b (b) 6 (6)	13.41 ± 2.13 a (a) 6 (6)	9.59 ± 1.50 ab (a) 6 (6)
stem		46.39 ± 6.53 b (b) 6 (7)	93.88 ± 5.5 b (b) 6 (7)	119.93 ± 6.14 ab (b) 6 (7)	14.85 ± 1.31 c (a) 6 (7)	11.32 ± 0.50 b (a) 6 (7)
branch	7.44 ± 1.07 a (a) 6 (7)	12.16 ± 1.66 a (a) 6 (6)	46.99 ± 1.42 a (a) 6 (6)	59.75 ± 2.54 a (a) 6 (6)	65.54 ± 1.65 c (b) 6 (6)	12.56 ± 0.70 b (a) 6 (6)
Seasonal						
Gliricidia sepium						
root	6.41 ± 1.29 a (a) 6 (8)	48.79 ± 12.49 ab (a) 6 (6)	81.05 ± 5.67 ab (a) 6 (6)	130.1 ± 8.35 b (b) 6 (6)	16.7 ± 2.84 ab (b) 6 (6)	10.85 ± 1.80 ab (ab) 6 (6)
stem		31.58 ± 4.82 b (a) 6 (6)	118.11 ± 3.85 c (b) 6 (6)	146.64 ± 5.76 b (b) 6 (6)	4.35 ± 0.3 b (a) 6 (6)	5.32 ± 0.42 a (a) 6 (6)
branch	5.54 ± 0.98 a (a) 6 (10)	19.44 ± 1.5 ab (a) 6 (6)	67.75 ± 1.88 b (a) 6 (6)	95.04 ± 2.41 b (a) 6 (6)	20.57 ± 1.65 a (b) 6 (6)	8.55 ± 0.52 a (b) 6 (6)
Leucaena leucocephala						
root	61.72 ± 22.77 a (a) 6 (6)	149.49 ± 32.24 b (b) 6 (6)	112.25 ± 6.54 bc (b) 6 (6)	158.51 ± 9.23 b (b) 6 (6)	16.4 ± 0.4 a (b) 6 (6)	19.93 ± 3.39 b (b) 6 (6)
stem		51.84 ± 22.93 ac (ab) 4 (4)	109.09 ± 8.14 bc (b) 4 (4)	159.85 ± 14.71 ab (b) 4 (4)	5.36 ± 0.4 b (a) 4 (4)	6.54 ± 1.43 ab (a) 4 (4)
branch	3.85 ± 0.97 a (a) 6 (6)	24.05 ± 1.99 b (a) 6 (6)	76.76 ± 1.03 c (a) 6 (6)	98.75 ± 2.24 b (a) 6 (6)	17.87 ± 1.11 a (b) 6 (6)	8.94 ± 0.64 a (a) 6 (6)
Erythrina subumbrans						
root	265.03 ± 87.86 b (b) 6 (8)	818.17 ± 181.89 c (c) 6 (8)	133.33 ± 10.11 c (b) 6 (8)	298.17 ± 22.34 c (b) 6 (8)	16.53 ± 1.97 a (b) 6 (8)	35.40 ± 1.65 c (c) 6 (8)
stem		111.45 ± 11.21 c (b) 6 (9)	194.54 ± 6.44 d (c) 6 (9)	265.14 ± 7.72 c (b) 6 (9)	1.71 ± 0.09 a (a) 6 (9)	5.82 ± 0.29 a (a) 6 (9)
branch	3.96 ± 1.42 a (a) 6 (7)	30.81 ± 4.2 b (a) 6 (6)	71.91 ± 4.99 bc (a) 6 (6)	110.6 ± 5.39 b (a) 6 (6)	22.09 ± 3.1 ab (b) 6 (6)	10.88 ± 0.82 ab (b) 6 (6)
